# Cardiac Hemangioma Mimicking Infective Endocarditis

**DOI:** 10.3390/diagnostics14192109

**Published:** 2024-09-24

**Authors:** Ching-Mao Yang, Yu-Ning Hu

**Affiliations:** Division of Cardiovascular Surgery, Department of Surgery, National Cheng Kung University Hospital, College of Medicine, National Cheng Kung University, Tainan 704, Taiwan; tarmo0515@gmail.com

**Keywords:** cardiac tumor, cardiac hemangioma, infective endocarditis, echocardiography

## Abstract

Cardiac hemangiomas are rare and often misdiagnosed due to their nonspecific clinical presentations. We report a case of a 70-year-old man presenting with chills and cold sweats, initially suspected of having infective endocarditis based on echocardiographic findings of a mobile mass on the mitral valve. Laboratory results showed leukocytosis and elevated C-reactive protein, but blood cultures were negative. Transesophageal echocardiography later revealed a well-defined mass with characteristics suggestive of a tumor. Surgical excision confirmed the diagnosis of hemangioma. Postoperative recovery was uneventful, with no mitral regurgitation. This case highlights the importance of considering cardiac tumors in the differential diagnosis of intracardiac masses.

Cardiac hemangiomas are rare cardiac tumors usually described in case reports [1]. Cardiac hemangiomas usually lack significant clinical manifestations unless cardiac compression or obstruction occurs [2]. Herein, we present a case of cardiac hemangioma initially misdiagnosed as infective endocarditis based on the imaging and clinical presentation.

A 70-year-old man presenting with chills and cold sweats was transferred to the emergency room. An echocardiogram revealed a mobile 1.57 × 2.47 cm mass on the mitral valve (Figure 1A,B); consequently, vegetation was suspected (details in Supplementary Table S1). The laboratory tests revealed leukocytosis, elevated C-reactive protein levels, and negative blood culture (details in Supplementary Table S2). However, the echocardiologist questioned the diagnosis because of the mass’s smooth, well-defined shape (differential diagnosis in Supplementary Table S3). Transesophageal echocardiography identified a 1.9 × 1.4 cm polypoid mass with a pedicle attached to the annulus near P2, suggesting a tumor (Figure 1C,D). During surgery, a round 1.5 × 1.5 × 1.5 cm tumor was identified at the mitral annulus between P1 and P2 that was connected to underlying mitral annulus calcification (Figure 2A,B). The tumor and a small part of the annulus endocardium were excised, and a mitral ring annuloplasty was performed. Pathology confirmed the diagnosis of hemangioma with dense lymphocyte infiltrate (Figure 2C,D). Postoperative echocardiography revealed no mitral regurgitation. Previous reports have mentioned that hemangiomas on the mitral valve may cause multiple brain infarcts [3]. Other types of intracardiac tumors, such as cardiac papillary fibroelastoma, have also been misdiagnosed as infective endocarditis [4]. Through this case, we hope to offer clinicians with additional perspectives for the diagnosis and management of intracardiac mass lesions.

## Figures and Tables

**Figure 1:**
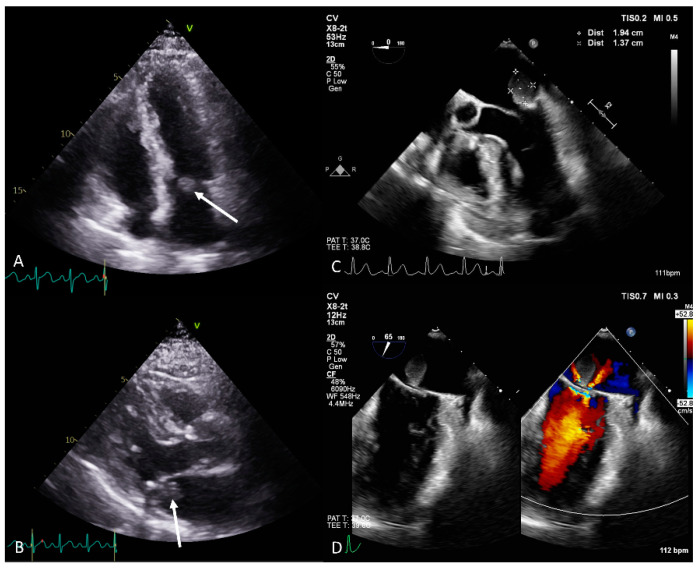
(**A**) Four-chamber transthoracic echocardiography view showing a mobile mass (white arrow) on the posterior leaflet of the mitral valve. (**B**) Parasternal long-axis transthoracic echocardiography view showing a mobile mass (white arrow) on the posterior leaflet of the mitral valve (Video S1). (**C**) Transesophageal echocardiography showing a 1.9 × 1.4 cm polypoid mass with a pedicle attached to the annulus near the P2 segment. The mass partially protruded into the left ventricle during diastole (Video S2). (**D**) Transesophageal echocardiography showing a polypoid mass with a pedicle attached to the annulus near the P2 segment. Color Doppler indicated mild mitral regurgitation. Grading of the mitral regurgitation may have been underestimated due to the mitral mass obstacle (Video S3). Preoperative coronary artery angiography showed no obvious feeding arteries (Videos S4–S6).

**Figure 2:**
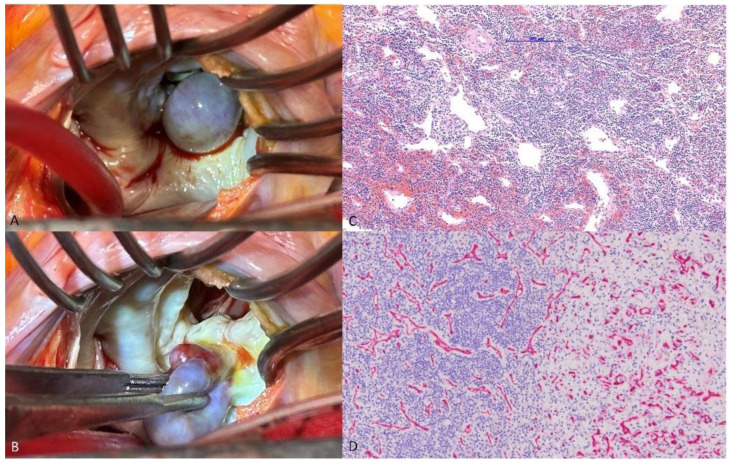
(**A**) A round 1.5 × 1.5 × 1.5 cm tumor at the mitral annulus between P1 and P2. (**B**) A tumor on the posterior mitral annulus connected to underlying mitral annulus calcification. (**C**) Pathology showed hemangioma with dense lymphocyte infiltration (5× magnification). (**D**) CD34 stain revealed a capillary–cavernous hemangioma with dense lymphocyte infiltration (10× magnification).

## Data Availability

The data are available on request from the authors.

## Supplementary Materials

The following supporting information can be downloaded at https://www.mdpi.com/article/10.3390/diagnostics14192109/s1. Supplementary Table S1. Details of patient’s echocardiography. Supplementary Table S2. Details of patient’s laboratory data. Supplementary Table S3. Differential diagnosis of the intracardiac mass in this case. Video S1. Parasternal long-axis transthoracic echocardiography view showing a mobile mass on the posterior leaflet of the mitral valve. Video S2. Transesophageal echocardiography showing a 1.9 × 1.4 cm polypoid mass with a pedicle attached to the annulus near the P2 segment, partially protruding into the left ventricle during diastole. Video S3. Transesophageal echocardiography color Doppler showing a mild mitral regurgitation jet. Grading of mitral regurgitation may have been underestimated due to the mitral mass obstacle. Video S4. Coronary angiography for left anterior descending artery. Video S5. Coronary angiography for left circumflex artery. Video S6. Coronary angiography for right coronary artery.

## References

[1] Li W., Teng P., Xu H., Ma L., Ni Y. (2015). Cardiac hemangioma: A comprehensive analysis of 200 cases. Ann. Thorac. Surg..

[2] Val-Bernal J.F., Terán-Villagrá N., García-Diego O., Sarralde J.A. (2017). Lymphocyte-rich capillary-cavernous hemangioma of the mitral valve: A case report and review of the literature. Cardiovasc. Pathol..

[3] Parkash O., Ying G.W., Ram A., Vemireddy L.P., Zahra F. (2021). A rare case of cavernous hemangioma of the mitral valve presenting as multifocal embolic brain infarcts. Cureus.

[4] Collado-Rivera C.J., Vojniku K., Sharma M., Fernandez H.A., Kaell A.T. (2023). Cardiac papillary fibroelastoma: Atypical presentation mimicking infective endocarditis with false positive commensal blood cultures. Cureus.

