# Supplementary material for: Cardiac Hemangioma Mimicking Infective Endocarditis
Source: Diagnostics (Basel). 2024 Sep 24;14(19):2109. doi: 10.3390/diagnostics14192109 (PMC11475683; doi:10.3390/diagnostics14192109)
Supplement: Supplementary file 1 [file diagnostics-14-02109-s001.zip › Supplementary Table.pdf]

Supplementary Table S1. Details of patient's echocardiography

	Data
Left atrium diameter	3.66 cm
Interventricular septum diameter	0.78 cm
Left ventricular end-diastolic dimension	3.87 cm
Left ventricular end-systolic dimension	2.23 cm
Left ventricular end-diastolic volume	56.98 ml
Left ventricular end-systolic volume	16.68 ml
Left ventricular ejection fraction	70.73 %
MV pressure half time	63.98 ms
MV Vmax	1.66 m/s
MV Vmean	1.02 m/s
MV maxPG	11.06 mmHg
MV meanPG	4.93 mmHg
MV velocity time integral	20.23 cm
MV area	3.37 cm^2
TR Vmax	1.514 m/s
TR maxPG	9.163 mmHg
Heart rate	115 bpm
Left ventricle stroke volume	68.17 ml
Left ventricle stroke volume index	35.88 ml/m^2
Left ventricle cardiac output	7.87 L/min
Left ventricle cardiac index	4.14 $L/min/m^2$

Abbreviation: MV, mitral valve; PG, pressure gradient; TR, tricuspid regurgitation.

Supplementary Table S2. Details of patient's laboratory data

	Data	units
Hemoglobin	12.7	g/dL
Platelet count	497000	µL
White blood cell count	17000	µL
Neutrophil-Seg	80.5	%
Eosinophil	4.0	%
Basophil	0.7	%
Monocyte	7.9	%
Lymphocyte	6.9	%
Glucose (random)	196	mg/dL
Potassium	3.8	mmol/L
Sodium	135	mmol/L
Serum Creatinine	0.69	mg/dL
Aspartate aminotransferase	21	U/L
Alanine aminotransferase	22	U/L
Total bilirubin	0.4	mg/dL
NTproBNP	82	pg/mL
High sensitivity troponin T	11.3	ng/L

Supplementary Table S3. Differential diagnosis of the intracardiac mass in this case

Diagnosis	Diagnostic criteria/ tools	Consistent with the diagnosis	Not consistent with the diagnosis
Infective Endocarditis	Modified Duke's criteria, blood culture, echocardiography (TTE and TEE), FDG-PET/CT, cardiac CTA	Newly identified mass on the mitral valve	Negative blood cultures, absence of predisposing conditions, no fever, no embolic events, or immunologic phenomena
Fresh Cardiac Thrombus	Echocardiography (TTE and TEE)	None	Adequate left ventricular function, no history of arrhythmia, no systemic embolic events
Organized Thrombus	Echocardiography (TTE and TEE)	None	Adequate left ventricular function, no history of arrhythmia, clean left atrial appendage
Primary Cardiac Tumor			
- Myxoma	Echocardiography (TTE and TEE)	Occurs in the left atrium, typically in the endocardial layer, predisposing age: middle-aged	The tumor has a relatively regular shape and dense content, not irregular or jelly-like, no systemic embolic events
- Papillary Fibroelastoma	Echocardiography (TTE and TEE)	Occurs on cardiac valves, endocardial layer	No "sea anemone" appearance, no systemic embolic events
- Fibroma	Echocardiography (TTE and TEE)	More common in the myocardium	
- Lipoma	Echocardiography (TTE and TEE)	Typically found in the endocardial layer	Lipomas are more frequent in the right atrium and left ventricle

- Rhabdomyoma	Echocardiography (TTE and TEE), cardiac MRI, cardiac CTA	None	Predisposing age: infants and children, more common in the myocardium
- Hemangioma	Echocardiography (TTE and TEE)	Occurs in the left atrium, endocardial layer, smooth, well-defined shape	None
- Leiomyosarcoma	Echocardiography (TTE and TEE), cardiac MRI, cardiac CTA	Occurs in the left atrium, endocardial layer	Leiomyosarcoma is more invasive, often involves pulmonary veins
- Angiosarcoma	Echocardiography (TTE and TEE), cardiac MRI, cardiac CTA	None	Angiosarcoma is more invasive, typically occurs in the right atrium, often involves the endocardium, myocardium, epicardium, and pericardium
Metastatic Cardiac Tumor			
- Metastatic Tumor	Echocardiography (TTE and TEE), cardiac MRI, cardiac/whole-body CTA	None	No continuity with major vessels, no history of malignancy
- Tumor Thrombus	Echocardiography (TTE and TEE), cardiac MRI, cardiac/whole-body CTA	None	No continuity with major vessels, no history of malignancy, more commonly occurs in the right atrium and right ventricle
Anatomic variants	Echocardiography (TTE and TEE), cardiac MRI, cardiac CTA	None	The mass was pedunculated from the mitral annulus and extended towards the mitral valve, but it was not part of the mitral valve or annulus
Artifacts	Echocardiography (TTE	None	Both TTE and TEE yielded consistent results, no

	and TEE), cardiac MRI, cardiac CTA		history of intravascular or intracardiac device use

Abbreviation: CTA, computed tomography angiography; FDG, F-18 fluorodeoxyglucose; MRI, magnetic resonance imaging; TEE, transesophageal echocardiography; TTE, transthoracic echocardiography.
